# Toward an architecture of attachment disorganization: John Bowlby’s published and unpublished reflections

**DOI:** 10.1177/1359104517721959

**Published:** 2017-08-09

**Authors:** Judith Solomon, Robbie Duschinsky, Lianne Bakkum, Carlo Schuengel

**Affiliations:** 1 Department of Public Health and Primary Care, Cambridge University, UK; 2 Adelphi University, USA; 3 Faculty of Behavioural and Movement Sciences, Vrije Universiteit Amsterdam, The Netherlands; 4 Clinical Child and Family Studies and Amsterdam Public Health, Vrije Universiteit Amsterdam, The Netherlands

**Keywords:** Attachment, Bowlby, disorganization, conflict behavior, fear

## Abstract

This article examines the construct of disorganized attachment originally proposed by Main and Solomon, developing some new conjectures based on inspiration from a largely unknown source: John Bowlby’s unpublished texts, housed at the Wellcome Trust Library Archive in London (with permission from the Bowlby family). We explore Bowlby’s discussions of disorganized attachment, which he understood from the perspective of ethological theories of conflict behavior. Bowlby’s reflections regarding differences among the behaviors used to code disorganized attachment will be used to explore distinctions that may underlie the structure of the current coding system. The article closes with an emphasis on the importance Bowlby placed on Popper’s distinction between the context of discovery and the context of justification in developmental science.

## Introduction

Infant disorganized attachment is a classification for the Ainsworth Strange Situation (Ainsworth & Wittig, 1969), used to identify behavior toward the caregiver that appears fearful, strongly conflicted, or disoriented. Since its introduction by Mary Main and Judith Solomon (1990), disorganization has become a matter of significant interest for researchers, clinicians, and policy-makers. This interest has been prompted by studies that report high rates of disorganized attachment in samples of maltreated infants, though the classification is also found at lower rates in community samples. Home and laboratory observations in both high- and low-risk samples show links between frightening, frightened, and dissociative caregiving behaviors and infant attachment disorganization (Hesse & Main, 2006; Madigan et al., 2006). Other researchers report associations with caregiver withdrawing behaviors (Lyons-Ruth et al., 2013) and with major separations between child and caregiver (Solomon & George, 2011). In turn, disorganized attachment predicts a number of negative outcomes, including well-replicated associations between infant disorganized attachment and later externalizing disorders (Fearon, Bakermans-Kranenburg, Van IJzendoorn, Lapsley, & Roisman, 2010). To date, disorganized attachment has been intensively studied, showing replicated links with a coherent set of precursors as well as outcomes, yet significant questions remain. The classification “undoubtedly identifies behavioural features of considerable theoretical and clinical significance, but the meaning of the pattern remains rather unclear” (Rutter, Kreppner, & Sonuga-Barke, 2009, p. 532). In this article, we will propose that the remarkable heterogeneity of behavioral indicators of disorganized attachment can best be understood in light of awareness of the historical and theoretical roots of the coding system.

In most cases, the behaviors identified by Main and Solomon (1990) as indicative of disorganization appear to be disruptions in or distortions of the well-known Ainsworth patterns. The classification process itself requires an evaluation of the intensity and duration of over 50 indexed behaviors patterns, divided into seven broad categories based on the morphology (appearance) of the behaviors. The theoretical “neutrality” of the compilation of behaviors, no doubt, has tended to support the general assumption by researchers and clinicians that the behaviors are essentially interchangeable as expressions of breakdown of the attachment system. However, some behaviors clearly reflect fear or apprehension, such as fleeing the caregiver, others suggest of dissociation, and the meaning of other behaviors is more obscure (Main & Hesse, 1992). Hesse and Main (2006) advise that it would therefore be “a worthwhile endeavor for developmental psychopathology” to study different caregiving contexts and “compare these to the forms of D behavior exhibited by their infants” (p. 335). Other researchers, representing clinical, developmental, and social psychological standpoints, have also made such calls (e.g. Beebe & Lachmann, 2014; Crittenden, 2016; Lyons-Ruth et al., 2013; Paetzold, Rholes, & Kohn, 2015; Slade, 2014; Zeanah & Gleason, 2010). To date, however, there is only limited research linking different disorganized behaviors to specific aspects of caregiving history. In a landmark paper, Padrón, Carlson, and Sroufe (2014) differentiated between infants classified as disorganized who showed frightened or disoriented behavior and those who did not. They suggested that the former group more often had parents who scored poorly on the Ainsworth Sensitivity/Insensitivity and Cooperation/Interference scales in the home, whereas infants in the latter group were more likely to have had difficulties with emotion regulation on the Brazelton Neonatal Behavioral Assessment Scale already in their first 2 weeks of life.

In the chapter on disorganized attachment in the latest edition of the *Handbook of Attachment*, Lyons-Ruth and Jacobvitz (2016) argue that sufficient data have now accumulated on disorganized attachment for a period of differentiation to begin. They propose the radical solution to the issue of the heterogeneity of indices of disorganized attachment of revalidating the construct based on those elements that predict later risk. In their chapter on measurement in the Handbook, Solomon and George (2016) also emphasize the need for further differentiation of the construct of disorganization. They suggest that researchers work toward the validation of a small number of scales to attend to possible distinctions within the overarching attachment category (cf. Abrams, Rifkin, & Hesse, 2006). Still another approach to differentiating the disorganized attachment construct would be atheoretical, using data mining approaches and factor analysis on large datasets to see what the data tell us about the underlying psychometric structure of the coding system. We know of several laboratories presently pursuing such work. However, the field lacks a theory or framework for conceptualizing differences among the indices of disorganization. This is likely to hamper the interpretation of findings; the design of the future studies of the relationship between caregiving, attachment, and later outcomes; and the flow of insights between clinical practice and basic research.

Our goal will be to outline a possible architecture of the phenomena of disorganized attachment, drawing distinctions among the behaviors captured by the classification. Simply put, our question is “How are the many different indices used to identify disorganization related to one another?” Naturally, the answer to this question will build on the decades of research and theory regarding the disorganized classification that have taken place since it was first proposed (Groh, Fearon, IJzendoorn, Bakermans-Kranenburg, & Roisman, 2017; Hesse & Main, 2006; Solomon & George, 2011). However, the focus of this article is to seek inspiration from a largely unknown source. With permission and encouragement from the Bowlby family, this article will explore discussions of disorganization contained in Bowlby’s unpublished texts housed at the Wellcome Trust Library Archive in London.^1^ The article will begin by situating Bowlby’s ideas in their original context of ethological discussions of conflict behavior, and especially the work of Hinde (1959, 1970). Bowlby’s specific reflections will then be explored regarding differences among the behaviors used to code disorganized attachment. The implications of these ideas will be considered for interpreting behavior shown in the Strange Situation, and for potential differences in their processes or cause. The article is intended, therefore, as simultaneously a contribution both to the history of the human sciences as well as to setting a research agenda with implications for attachment theory and clinical practice.

## Background

In the Ainsworth Strange Situation, infants are presented with two separations and reunions with their caregiver in a laboratory setting arrayed with interesting toys. Ainsworth, Blehar, Waters, and Wall (1978) reported three patterns of infant response. Most infants displayed distress on the departure of their caregiver, pleasure on reunion, and could be comforted by their attachment figure and returned to play. This was in line with Bowlby’s (1969b) concept of the “attachment system”—a behavioral system that develops in primate infants which activates the infant to seek the physical and attentional availability of their familiar caregiver under conditions of threat or distress. The attachment system, for Bowlby, was conceptualized as a distinct motivational system separate from other behavioral systems known to ethological and psychological research such as the fear, affiliation, and exploration systems. Ainsworth termed the attachment of infants who behaved in this way in the Strange Situation “secure.” Her home observations indicated that their caregivers had responded rapidly and appropriately to their cries and other signals of their desire for physical proximity and contact. A sizeable minority of infants, however, did not protest separation, turned and moved away upon reunion and appeared more interested in the toys than in their caregiver. Ainsworth termed these infants “avoidant”; her home observations suggested that they had learned from experience that their distress displays would be rebuffed. A third group of infants showed anger and were unable to be comforted on reunion even though they were clearly interested in contact with the caregiver; home observations showed that caregiver responses to these infants’ signals were delayed or unreliable. Due to the anger shown toward the caregiver, Ainsworth called this pattern of response “resistant.” Main (1990) theorized that avoidance and resistance were “conditional strategies” used to maintain the availability of a somewhat unresponsive and insensitive caregiver. Avoidance represented an implicit strategy for achieving a conditional proximity to a caregiver who discouraged close physical contact; resistance represented an implicit strategy for keeping the attention of the caregiver through alternations of anger and distress.

The “disorganized/disoriented” attachment classification was introduced as an additional category for the Ainsworth Strange Situation (Main & Solomon, 1986, 1990). The protocols for the new classification emerged from a close analysis of 200 video recordings of infants from both low- and high-risk samples whose behavior did not closely fit the Ainsworth patterns, suggesting disruption in the coordination of attention and behavior within the attachment system. Main and Solomon based the selection of index behaviors on the ideas of Robert Hinde, a noted ethologist and friend of John Bowlby, who was an expert in behaviors shown by animals experiencing conflict between incompatible motivational tendencies. Conflict was not the only criterion, however. Types of conflict that were already identified in the Ainsworth avoidance and resistance scales were parceled out; and in some cases, the conflict was not visible and had to be inferred, such as infants fleeing their caregiver on reunion. The behaviors were clustered into seven categories of indices, based on their morphology:

- Sequential displays of contradictory behavior,
- Simultaneous display of contradictory behavior,
- Undirected, misdirected, or incomplete movements,
- Stereotypies, mistimed movements, and anomalous postures,
- Freezing or stilling,
- Display of apprehension of the caregiver,
- Overt signs of disorientation.

Exemplars based on actual observations were listed in each category, resulting in over 50 indices in total. The resulting system amounts to an observationally based catalogue: The distinctions among these different forms of behavior into the I–VII categories were not explicitly based in theory but primarily in differences in how these behaviors looked. For example, gaze avoidance while approaching the caregiver, such as in “*infant approaches with head sharply averted”* and *“while in apparent good mood, infant strikes, pushes or pulls against the parent’s face of eyes”* are both placed in II (Simultaneous display of contradictory behavior). There is no implication that these events necessarily reflect the same qualities in the infant–mother relationship. In addition, no distinction was made among the indices with respect to classification as Main and Solomon (1990) argued that each was in some way the result of disruption of the attachment system by significant conflict or confusion, reflecting the child’s past experiences with the caregiver. In such an account, disorganization can be described as

the special conditions which arise when two or more incompatible innate or learned responses are elicited simultaneously. These are the conditions which have been widely referred to in the research and clinical literature as “conflict.” Under such conditions there may occur disorganisation of normal behaviour and the appearance of apparently non-adjustive responses.

But who is speaking here? In fact, the text comes from a discussion between the experimental animal psychologist Roger Russell (Chair of Psychology at University College) and John Bowlby (Tanner, 1953, p. 86) that occurred in 1952. At first sight, this is disorientating—or at least it was for us. The term “disorganization” in the context of attachment is generally associated with the Main and Solomon classification for the Strange Situation. However, theorizing about disorganization and attachment has a longer history, and one that may have particular value today. Main and Solomon (1990) were partly aware of this history—Bowlby’s (1980) attention to disorganization in Loss, for example, was mentioned in the chapters introducing the disorganized attachment classification. In print, Bowlby (1988) would commend the addition of the disorganized attachment classification, and he noted that the behaviors identified by Main and Solomon are likely “of great clinical concern” (p. 141). However, much of Bowlby’s earlier reflection on conflict behaviors and fear remained in unpublished notes on the other side of the Atlantic. Even for those who know Bowlby’s (1969b) work well, to date this missing context has left his published remarks on what would now be called disorganization—even a whole chapter in Attachment (Chapter 6)—difficult to interpret or use.

In the discussion that took place in 1952, Russell posed four questions to Bowlby about conflict between incompatible motivational responses: (1) “What is the etiology of different disorders of behavior produced by such conflict?” (2) “What factors affect susceptibility to such behavior?” (3) “What factors shape the kind of behavior shown?” and (4) “How is it possible to intervene?” (Tanner, 1953, p. 86). With reference to the Strange Situation, questions one, two, and four have received substantial empirical attention in the years since Main and Solomon introduced the disorganized classification. However, Russell’s third question—“What factors impact the kind of behavior shown?”—has too often been neglected. This is, essentially, a question about the immediate causation of the behaviors captured in the indices. Part of the value of asking about the factors that impact the kind of behavior shown is that explication at this level has the potential to improve our ability to isolate the behavior patterns with the greatest developmental and clinical significance, as well as help clarify an internal logic of the Main and Solomon indices to coders.

## The logic within conflict

Main and Solomon were explicitly applying the ideas of Robert Hinde from ethology to infant behavior in their introduction of the disorganized attachment classification. Bowlby, too, was deeply influenced by Hinde in thinking about the behavior resulting from incompatible motivational tendencies. In his work, *Animal Behaviour*, Robert Hinde (1966) identified several kinds of behavior that occur when an animal experiences conflicting or irreconcilable impulses or signals from the environment.

One type of conflict behavior was sequential display of the two tendencies. For example, he describes the conflict a male chaffinch may experience between a sexual tendency and fear of attack by the female; he suggests that this conflict for the male can be managed by inhibition during the act followed by quickly fleeing after copulation—while giving “the same call as that which is given in the presence of a flying predator” (Hinde, 1970, p. 250). A second kind of conflict behavior he called “compromise behaviour” in which an action occurs which expresses both tendencies simultaneously. For example, while stimulating aggression in Canada geese usually results in attack, and stimulating fear results in a tendency to flee, the stimulation of both at the same time results in threatening behavior by the geese (Hinde, 1970, p. 261). Such coordination of the two tendencies appeared readily functional to Hinde. However, he also observed expressions of conflict that did not appear functional but instead manifested as a patchwork of incomplete behaviors. For example, a threatened rhesus monkey may jerk forwards and backwards on the spot, showing the desire to attack and to flee while doing neither (see also Hinde, 1966, p. 286).

A third kind of conflict behavior identified by Hinde was forms of immobility or freezing which result from equal and simultaneous tendencies to approach and flee or withdraw. A final form of conflict behavior was the display of a seemingly irrelevant behavior, such as grooming or drinking, in response to equilibrium between conflicting tendencies to approach and flee. For example,

Suppose we train a rat to run up an alley for food, and then give it an electric shock at the goal box. When subsequently placed in the alley it may run a little way up . . . [then] hesitate there grooming its fur or cleaning its paws. (Hinde, 1970, pp. 246–247)

Hinde argued that the kind of activity that appeared was not random. In part, it was a response to cues in the environment. For example, when facing a conflict between a tendency to fight an opponent and a tendency to flee in fear, a turkey would “drink if water is available, but feed if food is there” (Hinde, 1970, p. 279). In brief, therefore, some forms of conflict identified by Hinde include sequential contradiction, simultaneous “compromise” contradiction, forms of simultaneous or sequential contradiction that are poorly coordinated, freezing, and irrelevant behaviors suggestive of tension (stereotypies).

What factors shape the kind of conflict shown? The behavioral results of motivational conflict are diverse and can have an “inexplicable” quality at first sight. Yet, the ethological community, including Hinde, agreed that they were neither random nor inexplicable, and that different forms of psychological process could indeed be tentatively supposed from differences in the behavior displayed. Tinbergen (1952) argued that “much of so-called ‘random behavior’ is not random at all” since “a given irrelevant act is often typical of a particular set of conditions” (1952, pp. 5–6). Similarly, Hinde (1954) argued from his observations of chaffinches that “the resultant behaviour depends not merely on the incompatible motor patterns, but on the nature of the competing tendencies,” (p. 316) and other ethologists supplied further evidence for this claim (e.g. Macfarland, 1966; Rosenblum & Harlow, 1963). Bowlby was in agreement with the position that the kind of conflict behavior shown was not random. In notes from January 1954, he gives the example that “the more frequently an activity is performed in the usual course of events, the more frequently do they appear” in the context of conflict. Arm-raising (for a coat to be put on) or hand-clapping will more likely appear for a child who has been taught to do this and the movement associated with some comfort. Aggressive behaviors or fleeing responses are predisposed in conflict situations when such behaviors are not unfamiliar (PP/Bow/H.226; Bowlby, 1956, p. 171).

Based on such regularities, he argues more generally that the form of behavior shown tends to be “specific . . . for a given conflict.” For Bowlby, conflict behaviors offer a window into determinate differences of psychological process (PP/Bow/H.226). The ethological perspective that variations in anomalous or “disorganized” behavior could be interpreted as a meaningful window into experience influenced attachment theory in the years immediately before Main and Solomon (e.g. Sroufe, Schork, Motti, Lawroski, & LaFreniere, 1984 on “unusual” and “disorganized” behaviors seen in the preschool classroom). The idea of a logic to at least some conflict or anomalous behavior is an important one to highlight from the start in this article, as it runs counter to widespread assumptions about disorganized attachment today that characterize it as interchangeable, random, chaotic behavior. Such assumptions are, we have observed (Duschinsky & Solomon, 2017), based in part on confusion of the technical and ordinary meanings of the term “disorganized.”

In May 1963, Bowlby organized a conference funded by the Medical Research Council. The conference, with the ethologists Tinbergen and Hinde as keynote speakers, had two topics. One was how to find lines of determinate difference within the variety of conflict behaviors; the other was to address the issue of “sensitive periods” for the development of behavioral systems. At the conference, Bowlby presented his own initial thinking on the first question, drawing on the observations made by ethologists of different kinds of conflict behavior. In his notes for the paper, Bowlby considered five different behaviors that had been observed by ethologists:

- Alternation between behavioral tendencies,
- Simultaneous contradiction between behavioral tendencies,
- A simultaneous contradiction in which some compromise is reached between the tendencies in behavior that expresses both,
- The redirection or misdirection of a tendency, and
- Displacement^2^ or stereotypic behaviors (PP/Bow/D.6/5).

Not only do these map to several of the Main and Solomon indices. They are even placed in the same order, presumably due to the common debt to Hinde. Whereas Main and Solomon had access to hundreds of recordings of infants displaying these behaviors in the Strange Situation, Bowlby had access primarily to ethological records, his clinical experience, and his own unusual powers of systemization. However, it is equally remarkable that Main was able to sort the behaviors under headings that have held up well over decades, and which would appear to be based to some degree on meaningful distinctions among caregiving histories (Padrón et al., 2014). In both cases, it was unquestionably Hinde’s work systemizing observations across dozens of species that lay the essential groundwork.

Bowlby argued, at the 1963 conference, that lines of determinate difference among conflict behavior are difficult but not impossible to discern. He would repeat this position across his later writings, both unpublished and published. Even in the context of motivational conflict, “provided observations are skilled and detailed, therefore, a record of the behavior of very young children can be regarded as a useful index of their concurrent mental state” (Bowlby, 1969b, p. 6). Specifically, Bowlby argued that relative degrees of pathology can be discerned from differences among conflict behaviors. For example, he considered forms of conflict behavior that might be more or less unfavorable. Some forms of alternation that retained environmental responsiveness, he suspected, might be functional in certain regards. In considering “Behavioural sequences deriving from both tendencies are exhibited,” Bowlby (1969b) argues of “alternating behaviour” that

In some cases behaviour deriving from both tendencies is exhibited in such a way that behaviour of one sort alternates with that of the other sort. Although it sounds as though the result would be unfavourable, it is by no means necessarily so. (p. 98)

One close reader of Bowlby was, of course, his colleague and friend Mary Ainsworth. In *Patterns of Attachment*, Ainsworth pinpointed several conflict behaviors observed in the Strange Situation whose underlying function or cause she wished to be identified, and proposed the use of physiological measures to shed further light on the matter. These included “alternation of behaviors,” “fragmentary behavioral representatives of one or the other system,” and “behavior activated by one stimulus object may be redirected towards another that is not involved in the conflict” (Ainsworth et al., 1978/2015, p. 273). Despite the canonical centrality of *Patterns of Attachment*, her discussion of differences among what would now be called disorganized behaviors has never been cited or mentioned in print nor has her suggestion that physiological measures might help discriminate among them. It has likely not been clear to readers that these behaviors mentioned in Ainsworth’s classic text refer to what would now be termed disorganized forms of attachment behavior.

## Revisiting the Main and Solomon indices

As we have seen, Bowlby was addressing conflict or disruption of the attachment system and its behavioral expressions already from the 1950s. He developed a variety of ideas, largely unpublished, about different forms of conflict behavior, including the extent to which they are a product of disturbance and the extent to which they represent pathology. Some of Bowlby’s reflections address behaviors—such as pervasive withdrawal from the caregiver, self-harm, or indiscriminate friendliness—that are of clinical interest but which do not figure within the Main and Solomon indices (Granqvist et al., 2017). For the sake of scope and coherence within this article, we leave these to the side. Among the behaviors used by Main and Solomon in operationalizing disorganized attachment, Bowlby’s reflections suggest four clusters which summarize his ideas, allowing us to compare these with the ideas of Main and Solomon. We number and place them in this order on the basis of the hierarchy of risk considered by Bowlby—though we would highlight that this is our numbering, not his:

- *Cluster 1*. Direct expressions of the fear behavioral system,
- *Cluster 2*. Disorientation,
- *Cluster 3*. Conflict behaviors without overt fear,
- *Cluster 4*. Stereotypies.

As we have seen, the latter two clusters were considered already in Bowlby’s 1963 MRC address and in the section “Incompatible behavioural systems: results of simultaneous activation” in Attachment (Bowlby, 1969b); the first two were elaborated as a consequence of attention paid by both Bowlby and Hinde to the fear behavioral system from 1968, provoked by dialogue with Gordon Bronson (1968). Key texts on the latter two clusters include “Types of fear response” from 1968 (PP/Bow/H.209), the chapter “Forms of Behaviour Indicative of Fear” in Separation (Bowlby, 1973), and the letter to Gordon Bronson of 11 April 1974 (PP/BOW/J.9/40). There is no expectation that these clusters form discrete, non-overlapping categories. In the handwritten marginalia on his personal copy of Main and Solomon (PP/Bow/J.7/6), Bowlby emphasized that the different indices identified by Main and Solomon will likely co-occur in particular infants seen in the Strange Situation. This did not mean, however, that they were interchangeable. Bowlby suspected that their differences may reflect qualitative differences in parent–child interaction or in the child’s processing of experiences with the parent.

Due to the common debt to Robert Hinde, the clusters of behavior in Bowlby’s account align closely with distinctions in Main and Solomon, permitting exploration of parallels and correspondences between the two conceptualizations. Cluster 1 parallels “direct indices of apprehension” in Main and Solomon (index category VI). Cluster 2 parallels “direct indices of disorientation” (VII). Cluster 3 parallels “sequential” and “simultaneous” contradiction without overt fear (I and II). Cluster 4 matches with Main and Solomon’s identification of “stereotypies” (IV). Main and Solomon used italicization to indicate those behaviors that most clearly indicated disruption of the attachment system; the frequency of italicizations becomes increasingly sparse as we descend through Clusters 1–4. Likewise, Bowlby discussed behaviors in these clusters as concerning to varying degrees: direct apprehension of the caregiver and disorientation were expected to be associated with greater risk; whereas, in Bowlby’s writings, stereotypies figure as an indicator of stress with numerous possible causes. One point that can be noted in support of such a hierarchy is that in Main and Solomon’s system direct expressions of fear or disorientation (VI and VII) were predominantly, though by no means exclusively, drawn from maltreatment and high-risk samples. This was mentioned in an earlier draft of Main and Solomon (1990), but was cut in the process of shortening an already lengthy manuscript. Crittenden (1988) also found an association between vigilant behavior toward their caregiver by toddlers in the Strange Situation and a history of physical maltreatment. More recently, and again in support, Padrón et al. (2014) reported differences in caregiving history between those children classified as disorganized who show direct expressions of fear or disorientation (VI and VII) and those who show neither of these indices. They, too, propose that these behaviors may represent a more concerning history of caregiver–child interaction, where the caregiver sometimes frightens the infant in some way (without this necessarily indicating abuse)—as opposed to other behaviors suggestive of conflict or bewilderment, perhaps more associated with a caregiver who has dissociative episodes following trauma.

In Table 1, we provide a crosswalk between Bowlby’s “clusters” and the Main and Solomon indices. When coding using the Main and Solomon indices, the same behavior can receive more than one code if it falls in multiple categories. For example, a child may show disorientation (VII) together with stilling (V), with certain logic to this association. In the crosswalk, in one column we have therefore identified the primary location that the cluster Bowlby identifies would fall in Main and Solomon.

**Table 1.** Crosswalk between Bowlby and Main & Solomon.

Bowlby	Primary location(s) in Main and Solomon^a^
Cluster 1. Direct expressions of the fear behavioral system	VI. “Direct indices of apprehension”
Cluster 2. Disorientation	VII. “Direct indices of disorientation” V. “Freezing & stilling” where this occurs without signs of vigilance
Cluster 3. Conflict behaviors without overt fear	I and II. “Sequential” or “Simultaneous contradiction” III. “Undirected/misdirected” where these are without direct signs of fear or disorientation
Cluster 4. Stereotypies	IV. “Stereotypies and anomalous postures”

a Roman numerals refer to the category of disorganized behavior in Main and Solomon (1990).

We will discuss each of these clusters of behavior in turn, drawing together history, theory, and observation. For each, we will begin by situating Bowlby’s attention to the behaviors, identify the key texts in which Bowlby discusses them, and draw links between Bowlby’s reflections and the kinds of behaviors described by Main and Solomon from observations of the Strange Situation.

## Cluster 1: fear

Bowlby argued that the attachment system is biologically channeled to predispose a child to approach his/her familiar caregiver when alarmed. Building from Bowlby, Main and Hesse (2006) proposed that a child’s experience of frightened, frightening, or dissociative caregiver behavior would be “one highly specific and sufficient, but not necessary, pathway to D attachment status” (pp. 310–311). The reason for this is that such caregivers would themselves become associated with fear for the child, producing a paradox or unresolvable conflict between approach and avoidance of which disorganized behavior could be expected to be the result. The Main and Hesse position, however, has been badly misinterpreted. It was not their intention to suggest that all the different forms of disorganized behavior represent fear in relation to the caregiver in the same way or to the same extent (see Main, Hesse, & Hesse, 2011).

Part of the problem is the ambiguity of the word “fear,” which Bowlby reflected upon after reading Bronson’s (1968) article, “The Development of Fear in Man and Other Animals.” Discussing Bronson’s work in his book Separation, Bowlby (1973, p. 114) warned that

The usual practice of including under a single heading, that of behaviour indicative of fear, forms of behaviour that have such different predictable outcomes is of great significance . . . It can very easily make for confusion . . . We are dealing, not with some single comprehensive form of behaviour, but with a heterogeneous collection of interrelated forms, each elicited by a slightly different set of causal conditions and each having a distinctive outcome. (See also Bowlby’s correspondence with Bronson, PP/BOW/J.9/40)

Only with this qualification firmly in view did Bowlby permit that, in his work, “fear behavior and feeling afraid are used as general-purpose terms, terms that encompass all forms of behavior and, for humans, all shades of feeling also. When greater discrimination is required, the terms used are freezing and withdrawal or escape behavior” (Bowlby, 1973, p. 121; see also Sroufe, 1979, p. 486).

The fear behavioral system and the attachment behavioral system in humans are generally well aligned: from infancy, we wish to escape *to* our attachment figures. Yet, Bowlby understood that fear could also present as conflict with respect to the caregiver. He was fascinated by ethological observations that if a male baboon saw a predator slowly approaching, the male may act themselves to scare the juvenile which would then quickly cling to them, despite also showing fear (Bowlby, 1969b, p. 227). Other examples from experimental work with animals supported this point:

Just as Fisher found that puppies follow the more persistently despite punishment, and Cairns found the same in lambs, so Harlow found that an infant monkey clings the more intensely in the face of punishment. In this experiment a cloth model was fitted with nozzles through which blasts of compressed air could be forced. A buzzer served as a conditioned stimulus that warned the infant of an impending blast, known to be a strong aversive stimulus to monkeys. Although the infant monkeys soon learned what to expect, instead of taking evasive action they did just the opposite. They clasped the model with increased vigour and so received on face and belly a blast at maximum intensity . . . This paradoxical behaviour is, of course, an inevitable result of attachment behaviour’s being elicited by anything alarming. (Bowlby, 1969b, pp. 215–216)

Bowlby speculated that even infants who experience physical violence from their caregiver would also be likely, ultimately, to approach the caregiver when alarmed:

A special but not unusual situation in which there is conflict between attachment behaviour and withdrawal is when the attachment figure is also the one who elicits fear, perhaps by threats or violence. In such conditions young creatures, whether human or non-human, are likely to cling to the threatening or hostile figure rather than run away from him or her. (Bowlby, 1973, p. 117)

Bowlby’s account would later be elaborated by Main and Hesse (1990): even when caregivers alarm their offspring, children are still “paradoxically” predisposed by the attachment behavioral system to seek in the caregiver the solution to this alarm. They propose six kinds of caregiver behavior that are likely to alarm an infant (Hesse & Main, 2006). One is overtly frightening behavior, such as threats, abuse, or aggressive behaviors without the metasignals of play. A second is frightened behavior displayed by the caregiver in the presence of the infant and in response to a source that is unidentifiable to the infant or which is in response to the infant him or herself (as for instance, a male child evoking frightened behavior in a mother who associates maleness with previous partner violence). The third kind of alarming behavior suggested by Hesse and Main (2006) are behaviors suggestive of a dissociative state, for example, where the “parent suddenly completely ‘freezes’ with eyes unmoving, half-lidded, despite nearby movement” (2006: p. 320). Hesse and Main also identify three kinds of behavior that suggest an altered state of consciousness in the caregiver, and which therefore may indirectly alarm the child: (1) if the caregiver shows any of the disorganized behaviors toward their infant (e.g. mistimed movements, approaching the infant with their head averted), (2) if the caregiver behaves in a timid-deferential way toward the infant, or (3) if the caregiver engages in sexualized behaviors toward their child.

Drawing on ethological observations, Bowlby made notes speculating that a special quality of the fear system is that it has evolved to be able to override any other behavioral systems for a time, without conflict (PP/Bow/H.65). This would elucidate the otherwise mysterious observation made by Main and Solomon (1990) that some children, with their attachment system activated in the Strange Situation, could nonetheless display a coherent fleeing response from the caregiver without the display of conflict that would be expected when two incompatible motivational systems were activated simultaneously. Bowlby (1969a) emphasized that “it is now known that young creatures show fear and take avoiding action without pain having played any part whatsoever” (p. 321). Robertson and Bowlby had observed children screaming and fleeing their caregiver in apparent fear on reunion after prolonged hospitalization, a period in which the attachment system would have been activated without assuagement for a long time; he advised parents not to worry if this happened with their child following a hospitalization (Bowlby, 1958, p. 9; see also PP/Bow/D.3/38). This is an important point for clinicians. It runs against quite a common misconception (criticized by Granqvist et al., 2016) that disorganization in the Strange Situation indicates maltreatment by the caregiver. Not only does disorganized attachment not serve as a specific marker for maltreatment, but Robertson and Bowlby had seen *manifest fleeing of the caregiver* shown by children whose only known trauma was major separation from their caregiver (Bowlby, 1973). This is an observation in urgent need of replication on samples of young children who have experienced major separation.

Overtly frightened infant behaviors define Main and Solomon’s VI (direct indices of apprehension). However, as Main and Hesse (1992, p. 169) observe, only those that are clearly and directly linked to the behavioral system were placed into VI, such as “fearful facial expressions”; others such as “freezing of all movement at parent’s entrance” would be coded in V (freezing/stilling) but “indirectly” express the fear system. Parents rarely are explicitly threatening during the Strange Situation session itself and frightening incidents involving the parent in the home may be remote in time. Therefore, what we observe is assumed to be a resonance of representational memory: if the child associates their caregiver with alarm then this creates a conflict between approaching and avoiding or fleeing in the Strange Situation. Though it sometimes occurs in at-risk samples, it is comparatively rare to see fear of the caregiver without attachment behavior, and thus fear responses are usually embedded in or combined in some way with attachment behaviors.

In Table 1, column 2 we noted indices in the Main and Solomon system (a system organized according to behaviors that look the same) that seem to directly agree with the Bowlby clusters (which differ by potential mechanism). Additionally, other behaviors may agree with Bowlby’s clusters more indirectly. However, such analysis raises an important consideration regarding freezing behavior. Where does this fit in relation to the distinctions drawn in this article? Main and Hesse (1992) refer to freezing as a direct expression of the fear behavioral system whereas later, influenced by Liotti (1992), they describe freezing as a form of disorientation suggestive of dissociation (Hesse & Main, 2006; Main & Morgan, 1996). However, looking closely at the examples used in these different chapters, we suspect that two different uses of the term “freezing” are in play. In Main and Hesse (1992), the descriptions are of vigilant freezing with a frightened facial expression. In Hesse and Main (2006), they are of freezing that appears confused or disoriented.

The distinction comes into focus if Bowlby’s attention to the freezing response is considered. In his discussion of the products of the fear system, Bowlby (1960a) noted that the function of escape is served by two main behavioral responses, fleeing and freezing, which can be activated by the perception of danger:

Fright, it is suggested, is the subjective experience accompanying at least two related instinctual response systems—those leading on the one hand to escape behaviour, and on the other to alert immobility or “freezing.” It is to be noted that as so defined it does not presuppose any conscious awareness of danger. Instead, it is conceived as being the accompaniment of certain instinctual response systems whenever they are activated. (p. 96)

Though both are possible outputs of the fear behavioral system, Bowlby theorized determinate differences between the fleeing and freezing responses. In his notes on “Types of fear response” from 1968 (PP/Bow/H.209) and then in Separation (Bowlby, 1973, p. 113), Bowlby speculated that the fleeing behavioral response would be associated with “frightened facial expression accompanied perhaps by trembling or crying, cowering, hiding, running away” to avoid the threat; by contrast, freezing might be associated with anxious, tense vigilance, and perhaps the startle reflex. Bowlby emphasized that a reason to distinguish fleeing from freezing was that “the conditions that elicit one form of fear behaviour may differ in certain regards from those that elicit another form” (Bowlby, 1973, p. 113). Bowlby was in intense discussion with Ainsworth over these matters in the early 1970s, and this led Stayton and Ainsworth (Bowlby, 1973, p. 227) to suggest that fleeing and freezing may well not be correlated with one another at high levels of activation, though both can be seen incipient in “wary” behavior. In Separation, Bowlby (1973, p. 154) noted ethological evidence that whereas fleeing was associated with a known threat, freezing was associated with the perception of an unknown threat, that is, a threat whose location and danger were not yet determined.

Behavior in the Strange Situation corresponding to Bowlby’s use of the term “freezing” is captured in Main and Solomon (1990) as a direct index of fear: *“Highly vigilant posture or appearance when in presence of parent. Movements or posture tense, infant gives the impression of being hyperalert to parent even or especially when parent is positioned behind her”* (p. 139). Such observations agree with the characterization by Kozlowska, Walker, McLean, and Carrive (2015) of freezing as “flight or fight on hold” (p. 267). However, behavior that is directly labeled “freezing” in Main and Solomon (V-freezing, stilling), is not defined as either tense or vigilant, but rather as *“the holding of movements, gestures, or positions in a posture that involves active resistance to gravity,”* for example, *“infant sits or stands with arms held out waist high and to sides*.*”* This behavior is probably closest to “tonic immobility,” a phylogenetically more ancient defense strategy that “appears to activate only when newer structures such as the amygdala are deactivated and when freezing and flight or fight are switched off” (Kozlowska et al., 2015, p. 9). In contrast to freezing in fear, where the function is to gather more information, “playing dead,” the common name for tonic immobility, has the adaptive function of suppression of fear and movement with the predictable outcome of being ignored by predators and is specifically activated by capture. Kozlowska et al. regard tonic immobility as expressing dissociative states such as derealization and depersonalization. This leads us now on to Bowlby’s attention to disorientation and dissociative processes.

## Cluster 2: disorientation

Disorientation had interested Bowlby from the beginning of his career, as he had seen this phenomenon in combat veterans (during his time as a military psychiatrist), evacuated children, and patients in his clinic. Even in the mid-1930s, in the research notes for his MD thesis (PP/Bow/D.2/46), he was offering speculations regarding its cause. Of particular importance for his thinking was the relationship with the caregiver in early childhood. A predisposition to disorientation in response to alarm would be expectable, for Bowlby, if there were obstacles or setbacks in the ability of the caregiver to serve as what he would later call “the psychic organiser” for the child, the one who “orients him in space and time” (Bowlby, 1951, p. 53). The paradigmatic case to which Bowlby always returned was Laura—the girl seen in Robertson’s famous film “A two-year-old goes to hospital.” Bowlby, Robertson, and Rosenbluth (1952) report that, during the hospitalization, Laura would initially appear not to recognize her mother, but “after a few minutes of blankness she ‘came to’ and responded to her real mother” (p. 86). Bowlby et al. (1952, p. 86) also document Laura’s return home. They report that she continued expressing her desire for reunion in a fixed way, even after her mother was in the room: “when her mother opened the door, Laura looked at her blankly and said, ‘But I want my Mummy’.” In undated notes from his filing cabinet, contained with other material from around 1955, Bowlby considers the role of dissociative processes in disorientation. His frame for thinking about this was the idea of fixed attention. Though not always present by any means, a frequent tell-tale marker of the kind of disorientation that interested Bowlby was that an individual would seem to “snap out of it,” which illustrates the fixedness of attention and potentially a dissociative state.

In the 1960s, Bowlby wrote in detail about dissociative and disoriented responses in unpublished manuscripts such as “Defences that Follow Loss: Causation and Function” (PP/Bow/D.3/78). His attention to such behaviors was provoked by his attempt to develop a general theory of defense mechanisms. He argued that all defenses arise in situations where some “disorganization” of mental processes threatens because of stress placed upon mental processes to the point that homeostatic processes are very costly or impossible (PP/Bow/D.3/78). However, defenses *themselves* enact a weakening of integration, in segregating away mental processes that may otherwise result in greater disorganization. Reflecting on the grief response, in which the attachment figure is necessarily gone forever, Bowlby (1961) theorized that one way that disintegration “is avoided, or at least partially avoided, is by an abrupt cleavage of the psychic apparatus of the kind Freud was studying at the end of his life” (p. 336). Forms of splitting—or what Bowlby called the “segregation” of mental processes—permit a certain resilience to the mental apparatus in the face of disintegrative threats precisely by accepting some determinate and limited degree of segregation, though they do disadvantage the organism in certain ways in the long-run. Avoidance, for example, achieves a limited segregation by diverting attention away from attachment cues and toward, for example, the world of physical objects; it rigidifies but does not in itself undermine organization. In contrast to avoidance, dissociation is a more emergency measure for Bowlby, enacting a greater segregation in response to a higher intensity of threat of danger or loss of an attachment figure.

Bowlby (1960b) speculated that cases of preoccupied or fixed attention directed away from its original object were particularly predisposed by experiences where the link between desire and its fulfillment had been chronically and painfully frustrated. He drew evidence for this view from ethology, where there had been varied observations that motivation could be severed from aim under such conditions (Bowlby, 1960a, p. 100; PP/Bow/D.3/1 and 38). Hinde, for example, had observed dissociative-like behavior and redirected attachment behavior in infant rhesus monkeys following chronic separations. He had also made note of such behavior when the caregiving system had been activated without assuagement, as for instance in rhesus monkey mothers when another female had kept their infant for several hours and would not give it back (Hinde, 1959; Hinde, presentation at Bowlby’s MRC Ethology Meeting, PP/Bow/D.6/5; Spencer-Booth & Hinde, 1967). However, Bowlby may have also been thinking back to the disorientation he had seen and written about in combat veterans during World War II. In a “Memorandum on War Neurosis,” written in 1940 with Kenneth Soddy, Bowlby observed that a large proportion of the soldiers showing signs of mental illness were also showing signs of confused disorientation. There, Bowlby and Soddy theorized that the disorientation was caused by a chronic activation of the soldier’s fear behavioral system but an equally chronic frustration of their desire to flee from battle (PP/Bow/C.5/1).

Some of the behaviors seen in the Strange Situation distinguish themselves by appearing overtly disoriented, whereas others do not. Main and Solomon set this out explicitly, in justifying why the classification is officially called “disorganized/disoriented.” Behavior directly suggestive of disorientation defines VII in Main and Solomon’s coding system. However, as Main and Morgan (1996, p. 108) and Carlson, Yates, and Sroufe (2008, p. 44) noted, behaviors indirectly suggesting disorientation appear elsewhere in the coding system, such as misdirected behaviors (III) where the child appears confused. Like Bowlby, Main emphasized dissociation as a possible mechanism for such behaviors. This is in contrast to other forms of disorganized behavior, where “dissociative processes need not be inferred when, for example, an abused infant shows signs of fear in smiling at the parent, or makes awkward, repeated stop-start approach movements towards her. These movements and expressions indicate conflict between approach and flight” (Main & Morgan 1996, p. 124). Main and Morgan identify several behaviors as especially indicating disorientation, with a potential dissociative mechanism in play:

One candidate for dissociated action consists in an episode of distress or angry behavior which appears without explanation or warning . . . in addition, some infants have been observed raising arms to the stranger (with whom they have already spent several minutes) with a bright greeting as the parent enters the room. (Main & Morgan 1996, p. 125)

Hesse and Main (2006, p. 334) developed the earlier discussion in Main and Morgan further, observing that “while many D behaviors identified as disorganized are unlikely dissociative, as hiding under the chair at the entrance of a clearly frightening mother, some D behaviors (chiefly trance-like behaviors and seemingly dissociated actions) do seem to fit a dissociative model.” In making this distinction, their reflections are aligned with Bowlby’s unpublished remarks on the topic. Padrón et al. (2014) place VI (direct indices of apprehension) together with VII (disorientation) together as a single group. For Hesse and Main, as for Bowlby, disoriented behaviors are unlikely to mean quite the same thing as direct expressions of the fear behavioral system. However, both would appear especially concerning forms of disorganization, and it may make sense to put them together for that reason. Since Main and Morgan’s proposals, the Minnesota Longitudinal Study of Risk and Adaptation have reported relevant empirical findings. They found that “prospectively, most infant disorganization is not related to manifest pathological dissociation, but, retrospectively, most dissociation in later development can be traced to attachment disorganization in infancy” (Carlson et al., 2008, p. 45). Haltigan and Roisman (2015) reported that there is no association between infant disorganization and later dissociative symptoms in the National Institute of Child Health and Human Development (NICHD) dataset, and interpreted this as a failed replication of the Minnesota findings. However, there is no necessary contradiction keeping in mind Bowlby’s hierarchy of risk in which disoriented-dissociative forms of disorganization are among the more concerning. With the NICHD sample a normative cohort, and the Minnesota sample subject to extensive adversity (50% were teenagers, some as young as 12 years; 42% of the mothers did not complete high school; 37% were malnourished), it is not clear that the dissociative forms of disorganization specifically would be expectable in the NICHD sample.

## Cluster 3: conflict behaviors without overt fear

The Main and Solomon indices began simply as a list of behaviors discrepant with the Ainsworth classifications. They were defined by exclusion. The idea that they represented some disruption of the attachment system solidified only over the course of the 1980s. The Main and Solomon categories were sorted based on what behaviors looked the same: Category I comprised any kind of sequential contradiction that is not visibly fearful (such as strong proximity seeking immediately followed by strong avoidance); II represents any kind of simultaneous contradiction that is not visibly fearful (such as approach of parent with head sharply averted); and III represents miscellaneous other forms of conflict or interference with the attachment response.

One of Bowlby’s most characteristic arguments across his career was that emotional conflict can have different kinds and different degrees of intensity, and that this will be substantially shaped by experiences of attachment. For Bowlby, conflict behavior could still be controlled and environmentally responsive so long as the motivational responses in conflict were not too strong, as incompatibility would increase at higher degrees of activation. He reflected in his early notes on Freud and Klein that loss of control could be signaled by behavior that is poorly coordinated and environmentally unresponsive (PP/Bow/D.1/2/11). In an unpublished text circulated to colleagues at the Tavistock in February 1958, Bowlby emphasized that

Conflicts can vary in their intensity, i.e. in the amount of energy spent by conflicting forces, the importance attached by these forces to certain issues, the “cost” of victory or defeat. Conflicts can also vary in their violence of expression, i.e. the militancy of the means chosen for expressing conflict. It is important to distinguish clearly between these two. (PP/Bow/H.67)

Ainsworth et al. (1978/2015) and Main (1981) emphasized the conflict between anger and attachment behavior underlying both the avoidant and resistant classifications. Solomon and George (2011) noted that these organized patterns appear when emotional arousal can be regulated and reconciled in an environmentally responsive way. Avoidant infants outwardly maintain composure through directing attention toward the environment and away from the caregiver and from their own distress and frustration. Resistant infants, on the other hand, are known for alternation between attachment behavior and anger. They are difficult to console during the 3-minute reunions of the Strange Situation, yet they rarely dissolve into extreme distress or extreme anger. Though the two insecure attachment patterns are associated with less desirable outcomes than the secure pattern (Groh et al., 2017; Sroufe, Egeland, Carlson, & Collins, 2009), this level of motivational conflict in the infant–parent dyad in the organized insecure patterns does not appear to present a risk for serious maladaptation, suggesting that it usually remains within tolerable bounds.

Yet, Bowlby (1969b) observed that, under conditions of intense conflict between the attachment system and other affects or states, the management of the “competing tendency may be unstable or inefficient and the result be alternating behaviour of a non-functional kind” (p. 100). Pursuing this line of thought, it is possible that differences in the intensity or duration of infant conflict behavior can be explained by corresponding dimensional differences in caregiver behavior. In her early papers on avoidance, Main (1981) proposed that anger and rejection by mothers of insecure-avoidant infants created paradoxical approach–avoidance conflict for which displacement activities provided sufficient relief. Somewhat later, when she was exposed to more high-risk families, she came to appreciate the difference between rejection leading to approach–avoidance conflict of levels manageable through avoidance and alarming caregiving leading to disorganization (for a history of this period see Duschinsky, 2015). Compared to manifest expressions of the fear behavioral system, conflict behaviors and forms of approach-avoidance that are not overtly fearful (index categories I–III) might reflect a child’s experiences of milder forms of threat or types of alarming behavior from their caregiver. This may include dissociative, timid/deferential (Hesse & Main, 2006), or grossly aversive behaviors (Main & Stadtman, 1981) by the caregiver. We underscore Hesse and Main’s (2006, p. 335) proposal that exploration of associations between particular forms of caregiving history and particular forms of conflict behavior in the Strange Situation would be of great interest. We would, additionally, also highlight the study suggested already by Ainsworth et al. (1978/2015, p. 273): that of examining the physiological correlates of differences in the intensity or duration of infant conflict behavior.

## Cluster 4: stereotypies

Stereotypies and other behavior fragments comprise the last of Bowlby’s clusters, and category IV in Main and Solomon (1990). Bowlby had first observed stereotypic behavior in the traumatized soldiers returning from the war. Among the symptoms associated with severe cases, he noted, “amnesias, confusional states, transient psychoses, anxieties, depressions, dreams and panic states, trance states, severe tics” (PP/Bow/C.5/1). However, tics and other stereotypic behaviors were a wide-ranging symptom in the clinical cases seen by Bowlby, not solely characteristic of traumatized individuals. In his 1940 essay on “The Influence of Early Environment in the Development of Neurosis and Neurotic Character,” Bowlby gives a case study of a young boy with a severe tic and obsessional symptoms that occurred especially around expressions of desire or anger. In line with wider psychoanalytic thinking about stereotypies and tics (e.g. Ferenczi, 1921), Bowlby (1940, p. 168) interpreted the tic and obsessional symptoms as expressions of psychological conflict between prohibitions held in place by the mother’s anxiety about the boy, and his anger at the demands she made on him. In his literature review of the effects of institutionalization for the World Health Organization (WHO), Bowlby (1951, p. 17) also documented sensory-motor stereotypies in institutionalized children.

Bowlby’s overarching conclusion is that stereotypies may mark tension or stress. He reports a personal communication from Mary Ainsworth that the infants classified as insecure in the Strange Situation, who experienced distress that was either not shown or not comforted by the caregiver, were much more likely than securely attached infants to show stereotypic movements at home in the final quarter of their first year (Bowlby, 1969b, p. 388). Ainsworth et al. (1978/2015, p. 235) would later report findings indicating that the most insecure infants in the Strange Situation were also those who showed the most stereotypies both at home and in the Strange Situation. Another line of findings related to repetition of the Strange Situation, Rowell and Hinde (1963) documented that infant rhesus monkeys did not become acclimatized to experimental separations from and reunions with their caregiver, but instead showed more distress and stereotypies each time; this finding would be replicated by Ainsworth when she repeated the Strange Situation 2 weeks later with the infants of her original sample to test for stability in the patterns shown (Ainsworth et al., 1978/2015, p. 221; see also Granqvist et al., 2016). Bowlby was fascinated that with the greater anxiety associated with repeating the Strange Situation, every one of the Ainsworth infants who had initially shown avoidance now went to their caregiver with a display of distress, but did so while showing stereotypic behavior marking the dysregulation of their attempts at inhibition (Mary Main, personal communication). Pursuing this fascination, in 1966, Bowlby wrote to Ainsworth about his attempt, unfortunately unsuccessful, to recruit a specialist in stereotypic behaviors to join his team at the Tavistock (letter of 14 January 1966, Mary Ainsworth papers at the University of Akron, M3168, folder 2).

Stereotypies are already recognized by Main and Solomon as ambiguous indices of disorganization. They were included as a disorganization index because they seemed to represent conflict about displaying anger or distress when approaching the parent or when in the parent’s arms. However, none are italicized markers, as it was known that stereotypies can reflect a variety of sources of stress or conflict (Sroufe, Stuecher, & Stutzer, 1973). Their use for coding disorganization has subsequently been challenged particularly by early childhood specialists because stereotypies are also characteristic of disorders on the autism spectrum (Pipp-Siegel, Siegel, & Dean, 1999; Willemsen-Swinkels, Bakermans-Kranenburg, Buitelaar, van IJzendoorn, & van Engeland, 2000). For this reason, stereotypies that are performed throughout the episodes of the Strange Situation are not—or at least should not be—used as a basis for a disorganized attachment classification (Rozga et al., in press). In light of the causal hierarchy Bowlby perceives between conflict and stereotypies, it can be expected that stereotypies may well appear along with behavior from the other clusters, but that appearing on their own they indicate tension or attempted self-soothing rather than disruption within the attachment system.

## Conclusion

To date, the disorganized attachment classification collects a number of behaviors that look quite different because all, to varying degrees, suggest strong conflict at the level of the attachment system. Pianta, Egeland, and Sroufe (1990) have argued that aggregation of related phenomena has significant advantages for prediction and statistical power, and should be a mainstay of scientific inquiry. However, they advise that it is also “relevant and researchable” (Pianta et al., 1990, p. 230) to examine the relative importance of the elements and their interrelations (see also Kriss, Steele, & Steele, 2013). With reference to disorganized attachment specifically, Hesse and Main (2006, p. 335) urged that it would be “a worthwhile endeavor” to consider “the forms of D behavior exhibited by their infants” and reflect on their origins. Padrón et al. (2014) made an important contribution to answering this call. Their position is that inquiry into differences among the disorganized behaviors does not require alterations in the disorganized attachment construct, which can continue to be used to support aggregative research. However, there are possible advantages for prediction, study design, and intervention if meaningful and replicable distinctions can be discerned within the overarching category.

Exploring the John Bowlby Archive at the Wellcome Trust in London, we were struck by Bowlby’s conviction that meaningful and useful differences could be discerned among conflict behaviors, and by the fact that he even organized a conference with this as one of the two themes. The relentlessly systematic quality of Bowlby’s thinking underpins his continued relevance today in thinking about disorganized attachment, permitting the history of science in this case to operate “as a continuation of science by other means” (Chang, 2004, p. 249). Rather than merely interchangeable and lacking meaning, Bowlby treated conflict behaviors as having a logic in the interplay of the attachment system, the fear behavioral system, and other affective states. Infants classified as disorganized may frequently show more than on such behavior. Indeed, this is why Main and Solomon originally did not attempt to create subgroups within the new classification (or dimension). However, fuzzy-boundaried clusters may nonetheless be discerned, and can still be meaningful and potentially useful (Rosch, 1987). A first cluster of behaviors discussed by Bowlby are expressions of the fear behavioral system. In the Strange Situation, behavior strongly organized by the fear behavioral system can sometimes appear without conflict, but most often appears in the Strange Situation in conflict with attachment behaviors. A second cluster of behaviors are those suggesting disorientation, which Bowlby theorized as a product of segregation of aspects of the attachment system, breaking attentional processes off from their usual role in the attachment system in cohering perception, affect, and behavior. A third cluster discussed by Bowlby is those behaviors that suggest interference with the attachment system but without overt display of fear. These correspond to Main and Solomon’s indices I–III. Finally, Bowlby treated stereotypies as rather distinct, since these behaviors were more a general indicator of tension and stress and may especially, as Bowlby et al. (1952) and later Ainsworth et al. (1978/2015) suggest, indicate attempts to self-regulate when avoidant defenses begin to fail.

In his remarks about the behaviors, Bowlby placed stereotypies, conflict without overt fear, dissociation, and direct apprehension of the caregiver along a spectrum of increasing degrees of concern. Frank fear of the caregiver can provoke any of these behaviors (Main & Hesse, 1990). However, stereotypies and conflict without overt fear likely also have a variety of other causes, many of which are less suggestive of risk. For example, Main and Stadtman (1981) propose that if the caregiver behaves in a strongly aversive way to the infant’s attempt to gain contact when distressed, this will produce an approach–avoidance conflict that the child cannot manage by simply engaging with a toy. That is, the infant will be too distressed by their own caregiver to use the avoidant conditional strategy. Overall, the type of disorganized behavior can be hypothesized to be a product of (1) the nature of the eliciting factor (e.g. pain, separation, loss), (2) the behavioral systems and affects that are activated, (3) the intensity of the infant’s arousal or alarm, and (4) moderating factors, including the infant’s prior experience of the consequences of expressing negative effect. The hierarchy suggested by Bowlby’s writings aligns quite well with the emphasis given the specific behaviors that were highlighted in italics in Main and Solomon. With Bowlby’s private theorizing largely unavailable to them, and attempting to be cautious, Main and Solomon arranged the indices by what behaviors looked the same, not by potential mechanism. Yet, there is a substantial degree to which the clusters in Bowlby map already existing divisions in Main and Solomon, and the hierarchy of risk which his writings suggest. We find in Bowlby, therefore, a theoretical position that provides an architecture of disorganized attachment, offering a possible logic and conceptualization for what to date has often been seen as mere chaos.

In closing, we wish to offer a brief caution regarding the interpretation and direct use of the ideas presented here. In *Conjectures and Refutations*, Karl Popper (1963) presented a distinction between “the context of discovery” and “the context of justification.” In the context of discovery, conjectures and theories are being generated, and such ideas can stem from a variety of sources; in the context of justification, these are then subject to testing and attempted refutation. Failure to recognize the distinction, in Bowlby’s (1988) view, resulted in serious problems. These are outlined briefly in his book, A Secure Base (Bowlby, 1988, p. 84), and in more detail in his unpublished writings and notes on the philosophy of science (PP/Bow/H.98). One result of confusion between the context of discovery and the context of justification was premature confidence in the results of single research studies, occurring in the context of discovery: another was the tendency to reify constructs in the context of justification, losing track of particularities and differences within workable concepts, and so “lumping” together relatively unlike cases. The hierarchy of risk suggested by texts in the Bowlby Archive is, we think, important and interesting as a theory, as is Bowlby’s conceptualization of disorganization in terms of incompatible behavioral systems and affects of relative intensity. Bowlby’s distinctions may be able to help practitioners with “clinical formulation” about the particular case they are seeing: when issues of classification are less pertinent, it may be useful to think about kinds and degrees of conflict of the attachment system (Granqvist et al., 2017). Bowlby’s reflections may also shed light on the close relationship between the resistant and disorganized attachment classifications. Considerably more research, however, is needed regarding the theoretical propositions explored here. Nonetheless, as well as a support for the future research and discussion, we hope that clinical practitioners and researchers will take away from this article the knowledge that disorganized attachment is unlikely to be a static, homogeneous category and to keep this in mind when translating research findings on disorganized attachment into practical applications.

## Notes

1. The second author is presently at work on making a number of these texts available to the public through an edited volume of previously unpublished works by Bowlby.

2. Bowlby also highlights that these processes had each been observed by psychoanalysts. In Freud’s (1909/2001) case study of the Rat Man, for example, the patient repeatedly reports alternation between behavioral tendencies (e.g. actions to harm or protect his fiancée) and misdirected activities (e.g. wishing to reimburse the wrong person for a debt incurred). The same term, “displacement,” had overlapping (though non-identical) usage in ethology and psychoanalysis, referring to activity that occurs when the disposition to enact another is blocked (see Tinbergen & van Iersel, 1947).
